# Health outcomes of a high fructose intake: the importance of physical activity

**DOI:** 10.1113/JP278246

**Published:** 2019-06-09

**Authors:** Luc Tappy, Robin Rosset

**Affiliations:** ^1^ Department of Physiology University of Lausanne Lausanne Switzerland; ^2^ Cardiometabolic Center Broye Hospital, Estavayer‐le‐lac Switzerland

**Keywords:** *de novo* lipogenesis, lactate production, gluconeogenesis, exercise metabolism, exercise recovery

## Abstract

Fructose metabolism is generally held to occur essentially in cells of the small bowel, the liver, and the kidneys expressing fructolytic enzymes (fructokinase, aldolase B and a triokinase). In these cells, fructose uptake and fructolysis are unregulated processes, resulting in the generation of intracellular triose phosphates proportionate to fructose intake. Triose phosphates are then processed into lactate, glucose and fatty acids to serve as metabolic substrates in other cells of the body. With small oral loads, fructose is mainly metabolized in the small bowel, while with larger loads fructose reaches the portal circulation and is largely extracted by the liver. A small portion, however, escapes liver extraction and is metabolized either in the kidneys or in other tissues through yet unspecified pathways. In sedentary subjects, consumption of a fructose‐rich diet for several days stimulates hepatic *de novo* lipogenesis, increases intrahepatic fat and blood triglyceride concentrations, and impairs insulin effects on hepatic glucose production. All these effects can be prevented when high fructose intake is associated with increased levels of physical activity. There is also evidence that, during exercise, fructose carbons are efficiently transferred to skeletal muscle as glucose and lactate to be used for energy production. Glucose and lactate formed from fructose can also contribute to the re‐synthesis of muscle glycogen after exercise. We therefore propose that the deleterious health effects of fructose are tightly related to an imbalance between fructose energy intake on one hand, and whole‐body energy output related to a low physical activity on the other hand.

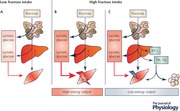

## Graphical abstract

Modulation of fructose metabolism by fructose intake and muscle energy output. In conditions of low fructose intake (*A*), available data suggest that fructose is primarily metabolized in the gut and, to a lesser extent, in the liver. Fructose metabolized in these organs then recirculates as glucose and lactate intermediates to be distributed to the periphery. With increasing fructose intake (*B*), intestinal fructose metabolism becomes saturated and fructose is mostly extracted by the liver where it is converted into metabolic intermediates. When total energy output is high, fructose conversion into glucose and lactate remains the preferred, most energy‐efficient disposal routes as both intermediates can provide energy to working muscle. When total energy output is low (*C*), however, the mismatch between fructose input and energy output forces the diversion of some fructose into lipids. According to this model, fructose's deleterious effects on health would only appear in conditions of chronically high fructose intake associated with low physical activity. IHCL: intrahepatocellular lipids; TRL‐TG: triglycerides in triglycerides‐rich lipoproteins.

## Introduction

Fructose, a stereo‐isomer of glucose, is a monosaccharide that is present in fruits, vegetables, and nectars or nectar products such as honey. Restricted by climatic factors to certain periods of the year, fructose consumption by our hunter–gatherer ancestors was relatively low and limited to these natural sources (Pontzer *et al*. 2018). In the past three centuries, successive developments of trade and extraction processes led to a massive increase in the availability of sucrose or table sugar, a disaccharide made up of one molecule of glucose linked to one molecule of fructose, and thus to an increased consumption of fructose (Johnson *et al*. 2007). Starting from the 1960s, sucrose consumption has been partially replaced by high fructose corn syrup, which is a solution containing 42% or 55% free fructose (HFCS‐42 and HFCS‐55 respectively), the rest being mainly free glucose and some glucose‐oligosaccharides (Hanover and White, 1993). Total fructose consumption thus increased drastically during the nineteenth and twentieth centuries to represent currently about 10% of total energy intake, or close to 50 g/day, in most affluent countries (Vos *et al*. 2008).

Based on associations between HFCS consumption and obesity prevalence in the USA, it was initially proposed that fructose may be causally linked to the development of obesity by increasing total energy intake (Bray *et al*. 2004). This hypothesis was shortly followed by proposals that fructose consumption could also be linked to the development of disorders such as diabetes, cardiovascular diseases, cancer or gout, independently of its effects on body weight (Nakagawa *et al*. 2006; Richelsen, 2013). A causal role of fructose in the pathogenesis of human non‐communicable diseases remains disputed, however, with several mitigating factors having been pointed out in recent systematic reviews (Wang *et al*. 2012, 2014). In the absence of a consensus, public health measures such as taxation of fructose‐containing sweeteners have been preventively adopted in several countries, with the premise of reducing fructose consumption (Bes‐Rastrollo *et al*. 2016).

Endurance athletes consume high amounts of fructose during physical activity. For a few decades, sports nutrition guidelines have consistently recommended a high carbohydrate intake before, during and after exercise to meet working muscle energy demands (Jeukendrup 2014). Accordingly, professional cyclists competing in a 3‐week ultra‐endurance event were found to ingest on average 463 g/day simple carbohydrates, a large part of it being fructose (Saris *et al*. 1989). It has further been proposed that adding fructose as a key ingredient of sports drinks and gels may improve carbohydrate metabolism and muscle performance during exercise (Jeukendrup, 2010). Yet, whether the daily ingestion of hundreds of grams of fructose by professional cyclists, or of lower but above‐average doses by people involved in intense recreational physical activity, can be detrimental to health remains unknown. This narrative review will thus draft an overview of fructose metabolism, focusing on its potential detrimental effects for health on one hand, and on its potential beneficial effects during exercise on the other. It will finally propose a simple model to account for the interactions of dietary fructose intake and physical activity on fructose‐related cardio‐metabolic risk factors.

## Overview of fructose metabolism

Contrarily to glucose, fructose is not readily phosphorylated by classical hexokinases, and requires for its initial metabolic steps the presence of a set of specific enzymes (fructokinase‐C, aldolase‐B and a triokinase) which convert it, first into fructose‐1‐phosphate, and then into triose phosphates (dihydroxyacetone phosphate and glyceraldehyde‐3‐phosphate, which are regular glycolytic intermediates). This set of ‘fructolytic’ enzymes appears to be solely expressed in small bowel enterocytes, hepatocytes and kidney proximal tubular cells (Mayes, 1993). Interestingly, the same cells also express gluconeogenic and lipogenic enzymes, can release glucose into the blood by the presence of glucose‐6‐phosphatase, and can also release lactate into the systemic circulation (Tappy, 2018). Hence, fructose metabolism takes place in cells that are also capable of releasing secondary intermediary metabolites into the blood.

**Figure 1:**
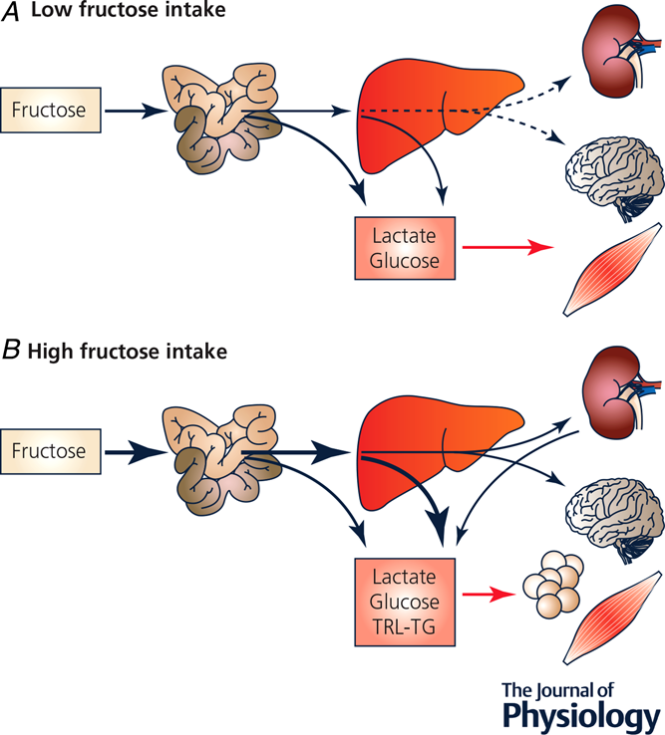
overview of fructose disposal pathways according to the amount of fructose ingested Rodent studies indicate that with small oral loads (*A*), almost all ingested fructose is taken up in small bowel enterocytes to be released into the blood as glucose, lactate and various other metabolites. Under such conditions, portal fructose concentration, and hepatic and systemic fructose metabolism are very low. With larger fructose loads (*B*), intestinal fructose uptake is most likely saturated, and fructose is delivered into the hepatic portal blood, from which it is largely extracted by the liver. Under such conditions, both the gut and the liver release fructose carbons as glucose, lactate and triglyceride‐rich lipoproteins into the systemic circulation. A portion (*ca* 15% of a 30 g fructose load), however, escapes gut and hepatic uptake and reaches the systemic circulation. In humans, the maximal fructose load being taken up by the gut, and the relation between total fructose intake and systemic fructose appearance, remain still unknown. TRL‐TG: triglycerides in triglyceride‐rich lipoproteins.

The initial steps of fructose metabolism are generally considered to occur mainly in fructolytic organs, but relative contributions of the intestine, the liver and the kidneys to total fructose disposal remain presently incompletely elucidated (Fig. 1). Recent observations made in rats suggest that, when fructose intake is very low (<0.5 g fructose/kg body weight), nearly all fructose is extracted by the gut where it is mainly converted into lactate and glucose. With higher fructose intake, however, fructose concentrations dose‐dependently increase in hepatic portal vein blood (Jang *et al*. 2018). In Yucatan miniature swine, it was shown that gut fructose metabolism accounted for about 12% of a 1.5 g/kg fructose load (Bjorkman *et al*. 1984), but no similar data are yet available in humans. The portion of fructose not metabolized in the gut then reaches the hepatic portal circulation, from which it is largely extracted at first pass to be converted into lactate and glucose within the hepatocytes. Glucose and lactate are subsequently released into the systemic circulation to serve as energy substrate in other cells of the body. Part of the glucose newly synthesized, however, remains in the liver to replenish hepatic glycogen stores. Fructose also potently stimulates hepatic *de novo* lipogenesis, and may increase postprandial triglyceride‐rich lipoprotein (TRL‐TG) secretion and blood triglyceride concentrations (Chong *et al*. 2007). Fructolytic enzymes are highly active in the liver and are essentially unregulated, with the consequence that intrahepatic ATP stores can be transiently depleted due to the rapid phosphorylation of large fructose doses. This has been mainly observed when fructose was infused intravenously at doses ranging between 62.5 and 500 mg/kg body weight (Terrier *et al*. 1989) and was associated with the formation of intrahepatic AMP, to an increase in purine degradation, and eventually to an acute increase in blood uric acid (Abdelmalek *et al*. 2012). Interestingly, the ingestion of very large boluses of fructose (50–75 g) by healthy volunteers confirmed these results (Dagnelie and Leij‐Halfwerk, 2010; Bawden *et al*. 2016), but whether similar effects would be observed with lower fructose doses remains debated (Wang *et al*. 2012).

It is generally assumed that ingested fructose is essentially metabolized in the gut and the liver, and that only a small portion of it gains access to the systemic circulation. It has, however, been recently documented, using a dual fructose tracer method, that about 15% of a 30 g fructose load escaped first‐pass splanchnic uptake and appeared in the systemic circulation over the 4 h following its ingestion (Francey *et al*. 2019). The fate of this systemic fructose yet remains unknown. It is likely that it is in part metabolized in the kidneys, since this organ has been shown to account for some 20% of intravenously infused fructose (Bjorkman and Felig, 1982; Bjorkman *et al*. 1989). In these experiments, however, about 30–40% of infused fructose was not accounted for by splanchnic or renal uptake, and may have been directly metabolized by other, non‐fructolytic organs (Bjorkman *et al*. 1984). Interestingly, the membrane transporter GLUT5 and the isoform fructokinase‐A, which are both supposed to be specific for fructose, are present in a wide set of tissues not restricted to the splanchnic region (Tappy, 2018). Hence, it is also plausible that non‐fructolytic organs metabolize dietary fructose to some extent.

Contrarily to glucose, intracellular fructose uptake is mediated by facilitated membrane transporters, GLUT5 and GLUT2, which are not regulated by insulin. Furthermore, its conversion to triose phosphates proceeds also independently of the presence of insulin (Mayes, 1993). This insulin‐independent metabolism has been largely suspected to be responsible for some of the deleterious effects of fructose (Bray *et al*. 2004) and was cited as a mechanism for the acute effects of fructose on postprandial lipaemia (Chong *et al*. 2007). Yet, fructose is as a consequence readily oxidized even in insulin‐resistant subjects with obesity or type 2 diabetes (Simonson *et al*. 1988). This peculiar metabolism may thus confer some benefit for glucoregulation in insulin‐resistant subjects. A meta‐analysis of controlled feeding trials indeed reported that isocaloric exchange of fructose for other dietary carbohydrates improved long term glycaemic control assessed from blood glycated haemoglobin concentration in diabetic patients (Cozma *et al*. 2012).

## Effects of high fructose diets on health and health‐related markers

Epidemiological data tend to indicate a plausible link between fructose or sugar consumption and the development of non‐communicable diseases (Te Morenga *et al*. 2012; Te Morenga *et al*. 2014; Malik, 2017). However, the nature of these studies prevents them from assessing causality, which can only be demonstrated by intervention studies. Such interventions have been performed in various animal models, and demonstrate that addition of fructose or sucrose to drinking water or to solid foods leads to energy overconsumption and to the development of obesity, insulin resistance or diabetes mellitus, dyslipidaemia, hepatic steatosis and hyperuricaemia in rodents (Bizeau and Pagliassotti, 2005; Nakagawa *et al*. 2006) and non‐human primates (Bremer *et al*. 2011). This set of concomitant alterations is strongly reminiscent of the human ‘metabolic syndrome’, which is considered a major risk factor for non‐communicable diseases (Mottillo *et al*. 2010; Esposito *et al*. 2012). For obvious reasons, no similar long‐term overfeeding studies have been performed in humans, however, and the effects of fructose on weight gain thus remain unclear.

Several human intervention trials nonetheless allowed documentation of short‐term effects of high fructose diets on cardiometabolic health markers such as blood lipids, glucose homeostasis, or intrahepatic fat content. It has been well‐documented that consumption of a fructose‐rich diet can increase cardiovascular risk through elevated fasting and postprandial blood triglyceride concentrations, both in healthy subjects and in obese or diabetic patients (Bantle *et al*. 1992, 2000; Livesey and Taylor, 2008). The mechanisms responsible remain unknown, but are thought to involve a stimulation of hepatic *de novo* lipogenesis, an increased hepatic TRL‐TG secretion and an impaired extrahepatic TRL‐TG clearance (Chong *et al*. 2007; Tappy, 2018). These effects were mainly apparent when fructose was consumed as part of hypercaloric diets (Wang *et al*. 2014), but were also reported with weight‐maintenance, high fructose diets (Bantle *et al*. 2000; Egli *et al*. 2013). These effects cannot be unequivocally attributed to fructose *per se*, however, and have also been reported with a range of high carbohydrate diets (Parks and Hellerstein, 2000). It has indeed been well documented that *de novo* lipogenesis was increased after 6 days on a hypercaloric, high fructose diet (Faeh *et al*. 2005), but also after 5 days on a hypercaloric, high glucose diet (Schwarz *et al*. 1995), after 7 days overfeeding with maltodextrins (Acheson *et al*. 1988), or after 25 days on a weight‐maintaining diet containing 75% carbohydrate as glucose polymers (Hudgins *et al*. 1996). Hence, some of the effects of fructose on lipid metabolism may not be specifically related to fructose, but to excess carbohydrate content at large.

Other human studies reported that a high fructose diet impaired insulin‐induced suppression of hepatic glucose production, but not whole‐body insulin‐mediated glucose disposal (Dirlewanger *et al*. 2000; Faeh *et al*. 2005; Ter Horst *et al*. 2016). Since the latter mainly corresponds to glucose utilization in skeletal muscle, this indicates that fructose specifically impaired hepatic, but not muscle, insulin sensitivity. A recent meta‐analysis further indicated that this effect was observed with weight‐maintenance diets as well as with hypercaloric high fructose diets (Ter Horst *et al*. 2016). Some studies however reported that intramyocellular lipid concentrations were increased in healthy subjects overfed with sucrose (Surowska *et al*. 2019) or in siblings of type 2 diabetes subjects overfed with fructose (Le *et al*. 2009). It raises the concern that longer exposure to fructose may possibly impair muscle insulin sensitivity since increased intramyocellular lipid concentrations are thought to be associated with muscle insulin resistance (Gemmink *et al*. 2017). Thus, available evidence suggests that fructose indeed can affect glucose metabolism, mainly through an incomplete suppression of hepatic glucose production. Whether fructose can also affect peripheral glucose consumption, and the resulting long‐term consequences on health, yet remains unclear.

The hypothesis that fructose may promote fatty liver disease is supported by available human data showing that consumption of a high fructose diet increases intrahepatic fat concentrations (Lecoultre *et al*. 2013; Surowska *et al*. 2019). However, similar effects were also observed with short‐term glucose (Johnston *et al*. 2013; Lecoultre *et al*. 2013) or fat (Sobrecases *et al*. 2010) overfeeding trials, suggesting that rapid increases in intrahepatic fat concentrations may be more related to excess energy intake rather than fructose *per se*. Nonetheless, several trials observed that reduction of dietary sugar consumption was associated with significant decreases in intrahepatic fat concentration of overweight and obese subjects (Campos *et al*. 2015; Schwarz *et al*. 2017; Schwimmer *et al*. 2019).

At this stage, intervention studies thus provide solid evidence that excess fructose (and fructose‐containing sugars), associated with excess energy intake, can increase cardiometabolic risk. High sucrose or high fructose diets in animals are associated with severe hepatic and muscle insulin resistance, and result in the development of diabetes mellitus, non‐alcoholic fatty liver disease, and severe hypertriglyceridaemia. This clearly points to deleterious health effects of fructose. In contrast, in short‐term overfeeding experiments with healthy, normal weight human subjects, one observes highly significant, yet modest increases in fasting blood glucose and in 24 h blood triglyceride concentrations. Similarly, intrahepatic fat concentrations increase 2‐ to 5‐fold within a few days, but still remain within normal limits. Whether changes of that magnitude would increase the risk for cardiovascular and metabolic diseases in the long term remains disputable, however. The major difference between animal and human studies is of course duration of exposure to fructose. Rodents had long exposure relative to their lifespan and largely increased their energy intake and body fat stores; their metabolic phenotype therefore reflected both chronic exposure to fructose and obesity‐associated metabolic complications. In contrast, human studies are limited to relatively short periods of time for both ethical and practical reasons. Whether a longer exposure to fructose would also lead in humans to a progressive impairment of glucose and lipid homeostasis, or to changes in body weight or body composition, is thus still unknown.

## Fructose metabolism during exercise

Whole body net carbohydrate and lipid oxidation can increase severalfold during physical activity to allow the continuous replacement of ATP used for muscle contraction. Glucose, lactate and fatty acids are prime energy substrates during endurance exercise, since they can be readily taken up from the blood by skeletal muscle fibres or be released from intramyocellular glycogen and lipid stores (Romijn *et al*. 1993).

Several observations indicate that dietary fructose is used as an energy substrate during exercise. In humans, ^13^C‐labelled fructose ingested before and during exercise was indeed found to be rapidly oxidized to ^13^CO_2_ (Decombaz *et al*. 1985; Jandrain *et al*. 1993). Furthermore, the amount of energy thus released makes a substantial contribution to the total energy cost of exercise (Jandrain *et al*. 1993). This was confirmed in various experimental conditions in which between 40 and 100% of fructose loads was oxidized during endurance exercise (Rowlands *et al*. 2015). Compared to single carbohydrate types, the co‐ingestion of mixtures of glucose and fructose is also known to increase exogenous carbohydrate oxidation during exercise (Adopo *et al*. 1994). This may derive from an enhanced maximal rate of gut monosaccharide absorption, since glucose and fructose are absorbed by two distinct, saturable glucose transporters, SGLT‐1 for glucose and GLUT5 for fructose (Shi *et al*. 1995), or from additional mechanisms (Rosset *et al*. 2017a; Tappy and Rosset, 2017). At this stage, it is clear, however, that a large amount of fructose is oxidized during exercise, and that fructose is indeed well suited to supply energy for living, active organisms.

Fructose metabolism during exercise remains unclear. Using stable‐isotope labelled metabolic tracers, it was documented that fructose is converted, presumably in the liver, into glucose and lactate then released into the systemic circulation to be subsequently taken up by skeletal muscle as energy substrates (Lecoultre *et al*. 2010). Accordingly, arteriovenous differences revealed that such substrate fluxes between splanchnic organs and working muscle can account for 78% of fructose disposal during low‐intensity exercise (Ahlborg and Bjorkman, 1990). Additionally, a direct fructose oxidation by skeletal muscle has not been demonstrated, but cannot be discarded, given that sarcolemma membranes contain specific fructose transporter GLUT5 (Douard and Ferraris, 2008), and that skeletal muscles synthesize an isoform of fructokinase‐C, fructokinase‐A (Diggle *et al*. 2009). Considering that hexokinase affinity is lower for fructose than for glucose, which is also present in much larger concentrations in the blood, we previously argued that muscle fructose oxidation is likely minor during exercise (Rosset *et al*. 2017a; Tappy and Rosset, 2017). However, fructose was recently found to be efficiently converted to fructose‐6‐phosphate by muscle hexokinase in fructokinase‐deficient mice (Miller *et al*. 2018), questioning the extent to which muscle fructose oxidation can represent an important fate during physiological exercise conditions.

Fructose can also be used to replenish hepatic and muscle glycogen stores during the post‐exercise, recovery period (Ahlborg and Bjorkman, 1990; Van Den Bergh *et al*. 1996). Here again, it appears that muscle glycogen re‐synthesis is mainly fuelled by glucose and lactate released from splanchnic tissues (Ahlborg and Bjorkman, 1990; Rosset *et al*. 2017b,*c*). Thus, these observations clearly indicate that fructose can be used efficiently to support skeletal muscle activity. It is used by skeletal muscle, not only as glucose, but also as lactate formed in splanchnic organs. Lactate itself indeed makes a substantial contribution to muscle metabolism during exercise and in the recovery period, and may also confer some advantages in terms of muscle energy efficiency (Rosset *et al*. 2017a; Tappy and Rosset, 2017).

## Physical activity as a modulator of fructose's health effects

It has been known for a long time, not only that fructose can be efficiently used as an energy substrate for working muscle, but also that the metabolic effects of high fructose diets were modulated by the level of physical activity. Several studies have indeed documented that, in rats fed a high fructose or sucrose diet, exercise training abolished fructose‐induced stimulation of lipogenic genes in the liver, and prevented the development of insulin resistance, hypertriglyceridaemia and hepatic steatosis (Zavaroni *et al*. 1982; Wright *et al*. 1983; Fiebig *et al*. 1998; Botezelli *et al*. 2010). Of note, most studies documenting the adverse metabolic effects of a high fructose diet were performed in subjects maintained under strict sedentary conditions. In contrast, when healthy subjects performed daily exercises at moderate‐to‐high intensity (two daily 30 min sessions on an ergometric bicycle at 125 W (Bantle *et al*. 2000; Egli *et al*. 2013), or 60 min brisk walking per day (Koutsari *et al*. 2001)), the increase in blood triglyceride concentrations and the stimulation of hepatic *de novo* lipogenesis induced by high fructose or high sucrose diets were completely prevented. This clearly indicates that the effects of dietary fructose on risk markers of disease are modulated by physical activity. Unfortunately, due to the paucity of human studies in this field, it remains unknown whether these effects of exercise are primarily mediated by alterations of total energy balance (i.e. exercise preventing a positive energy balance) or depend on other exercise‐related factors such as the balance between fructose energy intake and muscle energy output.

This leads us to propose that the overall effects of dietary fructose on health are tightly dependent on the balance between fructose energy intake on one hand, and total energy output on the other hand. Hepatic fructose uptake is essentially dependent on portal fructose appearance, i.e. on dietary fructose intake. Hepatic fructolysis being not regulated by insulin or by cellular energy status, fructose conversion into triose phosphates is proportional to fructose intake, irrespective of hepatic or extra‐hepatic energy needs. When produced in amounts exceeding hepatic energy needs, the triose phosphates are channelled into lactate synthesis, gluconeogenesis, or *de novo* lipogenesis. Conversion of triose phosphates to lactate and glucose in splanchnic tissues is associated with little energy cost, and these pathways for fructose disposal are likely to be favoured to *de novo* lipogenesis and synthesis of triglycerides, in which 20–30% of fructose energy is lost as heat (Tappy *et al*. 2013).

**Figure 2:**
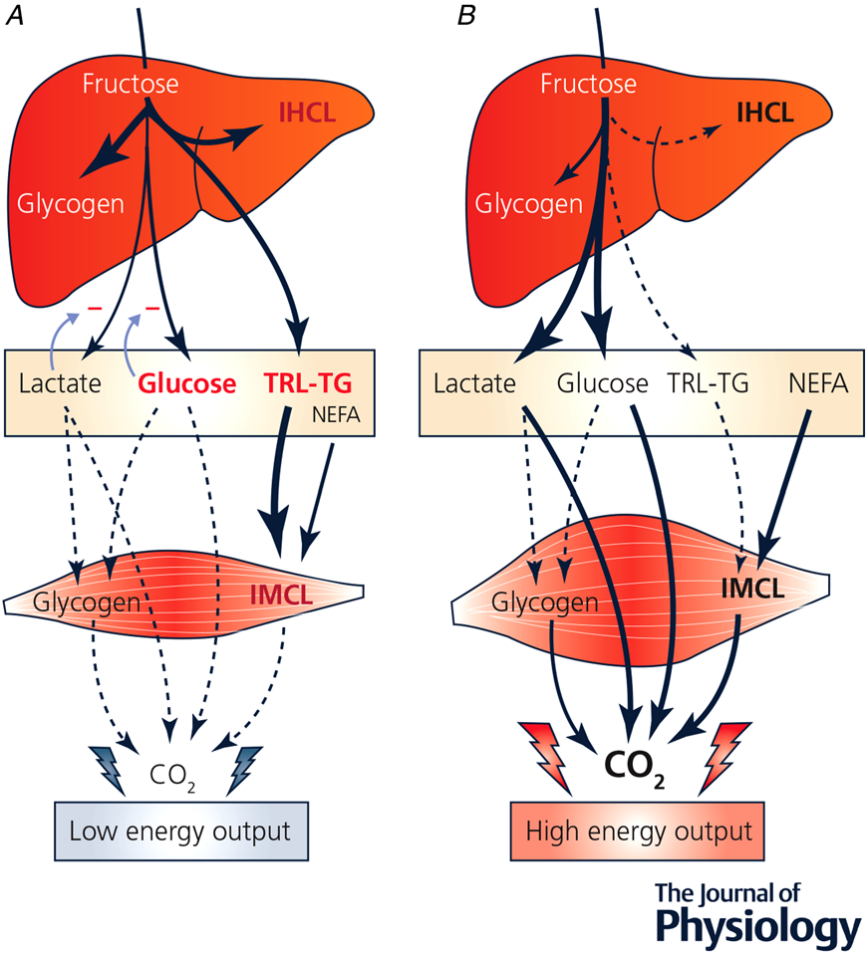
Proposed modulation of fructose metabolism by total energy output Fructose's metabolic effects may be largely dependent on the balance between fructose intake and whole‐body energy output. Hepatic fructose uptake is essentially proportional to portal fructosaemia, i.e. to dietary fructose intake. Intrahepatic fructolysis is not regulated by insulin or intracellular energy status, and hence intrahepatic production of triose phosphates is proportional to fructose intake. At high fructose intake, the effects of this large triose phosphate flux vary according to extrahepatic energy output. When total energy output is low (*A*), hepatic triose phosphate production exceeds hepatic energy need. Triose phosphates are first channelled into lactate and glucose synthesis to be released into the bloodstream. Since glucose and lactate utilization rates in resting muscles are low, blood glucose, lactate and insulin concentration tend to increase, and prevent a further increase in hepatic glucose and lactate release. The excess triose phosphates are then channelled into liver glycogen and intrahepatocellular lipid storage, and triglyceride‐rich lipoprotein secretion. This may in the long term lead to hepatic insulin resistance, non‐alcoholic fatty liver disease and dyslipidaemia. When total energy output is high (*B*), as during physical exercise, hepatic glucose production increases as a result of hyperglucagonaemia, and glucose and lactate uptake increase to meet the increased muscle energy needs. Release of fructose carbons as blood glucose and lactate proceeds at a high rate, while its storage as liver glycogen and intrahepatocellular lipids or its release in triglyceride‐rich lipoproteins remains quantitatively low. According to this model, which remains in part hypothetical, adverse health effects of dietary fructose would appear only when fructose intake chronically exceeds the capacity of the liver to release lactate and glucose for the periphery, i.e. mainly when there is a mismatch between fructose intake and muscle energy output. CO_2_, carbon dioxide; IHCL, intrahepatic cellular lipids; IMCL, intramuscular cellular lipids; NEFA, non‐esterified fatty acids; TRL‐TG, triglycerides in triglyceride‐rich lipoproteins.

With high fructose intake and low energy output, as in sedentary subjects (Fig. 2 *A*), we speculate that glucose and lactate release into the blood is inhibited by regulatory signals preventing hyperglycaemia and hyperlactataemia. There is indeed experimental evidence that hepatic gluconeogenesis is dose‐dependently stimulated by increasing intravenous fructose loads, but that hepatic glucose output is not (Surmely *et al*. 1999). This indicates that intrahepatic gluconeogenesis is tightly dependent on the rate of fructose administration, while hepatic glucose output is independently regulated by factors such as blood insulin and glucose concentrations. The mechanisms regulating hepatic lactate release remain largely unexplored, but one can speculate that hepatic lactate production associated with low extrahepatic lactate utilization will increase blood lactate levels, thus reducing the intra‐hepatic to blood lactate concentration gradient and the amount of lactate leaving the liver by facilitated diffusion. Under such conditions, fructose carbons may be initially stored as hepatic glycogen, then be diverted into *de novo* lipogenesis when hepatic glycogen stores become saturated, resulting in ectopic fat deposition in the liver, and increased triglyceride‐rich lipoprotein secretion.

In contrast, when fructose intake is high and energy output is high, as in physically active subjects (Fig. 2 *B*), hepatic release of glucose synthesized from fructose may increase due to glucagon production (Paquot *et al*. 1996; Surmely *et al*. 1999). Furthermore, glucose and lactate utilization rates increase in exercising skeletal muscle, thus preventing the development of hyperglycaemia and hyperlactataemia and hence inhibition of hepatic glucose and lactate output. According to this model, which remains in part hypothetical, adverse health effects of dietary fructose would appear only when fructose intake chronically exceeds the capacity of the liver to release lactate and glucose for muscle, i.e. mainly when there is a mismatch between fructose intake and muscle energy output.

## Conclusion

The available scientific literature regarding fructose health effects may appear somewhat confusing. On the one hand, there is accumulating evidence that prolonged excess fructose consumption may be linked to the development of non‐communicable diseases. On the other hand, many physically active humans remain healthy while consuming large amounts of fructose. The present review aimed to provide a potent reconciliation of this discrepancy, by delineating fructose metabolism in conditions of low or high fructose intake, together with low or high muscle energy output. The proposed mechanisms by which physical activity determines fructose health effects remain, however, largely hypothetical and will require further investigations.

Fructose metabolism may finally be considered from a historical perspective. In the pre‐industrial era, fructose ingestion from natural products occurred mainly during summer and fall, i.e. during periods in which energy storage was necessary in prevision of future shortage. Foraging activities and/or field work were, however, physically demanding tasks requiring an elevated muscle energy output. Thus, high levels of physical activity were almost inevitably present whenever fructose consumption increased during the year. Nowadays, however, the modern human lifestyle is associated with readily available foods and a low physical activity, and hence with a lower need of nutrients targeted for physical activity such as fructose. This imbalance may possibly explain the risks of adverse effects related to current fructose consumption.

## Additional information

### Competing interests

L.T. has received research support from Soremartec Italia Srl for projects unrelated to this report, and speaker's fees from Soremartec Italia Srl, Nestlé AG, Switzerland, and the Gatorade Sport Science Institute, USA. R.R. reported no conflict of interest.

### Author contributions

L.T. and R.R. drafted and edited the manuscript and figures. Both authors have read and approved the final version of this manuscript and agree to be accountable for all aspects of the work in ensuring that questions related to the accuracy or integrity of any part of the work are appropriately investigated and resolved. All persons designated as authors qualify for authorship, and all those who qualify for authorship are listed.

### Funding

This research was funded by a grant from the Swiss National Foundation to L.T. (grant no. 32003B_156167).
